# Treatment of bilateral mammary ptosis and *pectus excavatum* through the same incision in one surgical stage

**DOI:** 10.1590/S1516-31802012000300010

**Published:** 2012-07-12

**Authors:** Fernando Passos Rocha, Jefferson André Pires, Vinicius Franchini Torres, Djalma José Fagundes

**Affiliations:** I MD, MSc, PhD. Professor of Surgery, Universidade Católica de Pelotas (UCPEL), Pelotas, Rio Grande do Sul, Brazil.; II Medical Student. Universidade Federal de Pelotas (UFPel), Pelotas, Rio Grande do Sul, Brazil.; III MD, PhD. Associate Professor, Department of Surgery, Division of Operative Technique and Experimental Surgery, Department of Surgery, Universidade Federal de São Paulo (Unifesp), São Paulo, Brazil.

**Keywords:** Thoracic wall, Breast implants, Funnel chest, Surgery, plastic, Thoracic, Parede torácica, Implantes de mama, Tórax em funil, Cirurgia plástica, Cirurgia torácica

## Abstract

**CONTEXT:**

Congenital deformities of the anterior thoracic wall are characterized by unusual development of the costal cartilages. All these medical conditions are frequently associated with a variety of breast deformities. Several surgical techniques have been described for correcting them, going from sternochondroplasty to, nowadays, minimally invasive techniques and silicone prosthesis implantation.

**CASE REPORT:**

The present article reports the case of a young female patient who presented bilateral mammary ptosis and moderate *pectus excavatum* that caused a protrusion between the eighth and the tenth ribs and consequent esthetic disharmony. The proposed surgical treatment included not only subglandular breast implants of polyurethane, but also resection of part of the rib cartilage and a bone segment from the eighth, ninth and tenth ribs by means of a single submammary incision in order to make the scar minimally visible. Correction through a single incision benefited the patient and provided an excellent esthetic result.

**CONCLUSIONS:**

The techniques used to repair bilateral mammary ptosis and *pectus excavatum* by plastic and thoracic surgery teams, respectively, have been shown to be efficient for correcting both deformities. An excellent esthetic and functional result was obtained, with consequent reestablishment of the patient’s self-esteem.

## RESUMO

**CONTEXTO:**

Deformidades congênitas da parede torácica anterior são caracterizadas pelo desenvolvimento anormal das cartilagens costais. Todas essas afecções são frequentemente associadas com as mais diferentes deformidades da mama. Várias técnicas cirúrgicas têm sido utilizadas para a correção desde as esternocondroplastias até, atualmente, técnicas minimamente invasivas e próteses de silicone.

**RELATO DE CASO:**

O presente artigo relata o caso de uma paciente jovem que apresentava ptose mamária bilateral e uma forma moderada de *pectus excavatum* que ocasionava uma protrusão entre a oitava e a décima costelas, tendo como consequencia a desarmonia estética. O tratamento cirúrgico proposto incluiu, além de implantes mamários sub-glandulares de poliuretano, ressecção de parte da cartilagem da costela e parte do segmento ósseo da oitava, nona e décima costelas por uma única incisão sub-mamária para tornar a cicatriz minimamente visível. A correção por meio de uma única incisão beneficiou a paciente, proporcionando excelente resultado estético.

**CONCLUSÕES:**

As técnicas utilizadas para reparação da ptose mamária bilateral e do *pectus excavatum* pelas equipes de cirurgia plástica e torácica, respectivamente, demonstraram-se eficazes para correção de ambas as deformidades. Foi obtido ótimo resultado estético e funcional com consequente retomada da auto-estima da paciente.

## INTRODUCTION

Congenital deformities of the anterior thoracic wall are characterized by unusual development of the costal cartilages. The cartilage overgrowth causes the sternum to protrude forward (*pectus carinatum*), or push the sternum down (*pectus excavatum*).^1^ Its incidence rate is one case in every 300 people and its origins are still not completely known.^1^

The anatomical features presented may include prominence of the costosternal junction, torsion and rotation of the ribs, anterior or posterior projection of the costal gristle and associations with other conditions. All these medical conditions are frequently associated with breast defects.^2^

The current treatment for slight or moderate deformities of the thoracic wall is clinical follow-up and monitoring of the complications that may develop. Surgical treatment is usually performed only in cases of severe deformities.^3^

Several surgical techniques have been used since sternochondroplasty, which was created by Meyer (1911) and Sauerbrush (1913). This was followed by the surgical technique described by Ravitch in 1949, which gained worldwide recognition. This consists of resection of the aberrant costal arch while preserving the periosteum and elevating the sternum through using several biocompatible materials such as shafts and non-absorbable mesh.^4^

Today, minimally invasive techniques have been developed, including techniques using videothoracoscopy (Nuss), silicone prosthesis implantation (Marks), videoendoscopy (Kobayash) and sternal lifters (Onishi and Maruyama).^3^

The present article reports the case of a young female patient who presented moderate *pectus excavatum* that caused a protrusion of the thoracic wall between the eighth and tenth ribs, associated with bilateral hypomastia with consequent esthetic disharmony.

## CASE REPORT

**Figure 1.**
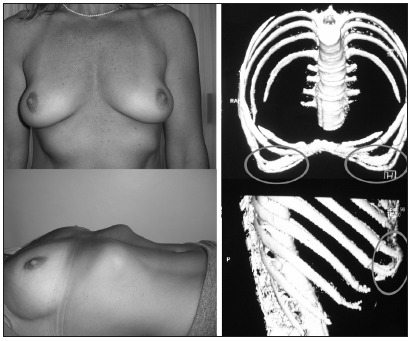
Photographs of the patient and multi-slice helical computed tomography before the operation.

A 35-year-old female presented with a moderate form of *pectus excavatum* that involved the lower edge of the chest bilaterally, including the area from the eighth to the tenth ribs. This was associated with bilateral hypomastia and, together, these presented esthetic deformity. After routine preoperative examinations, multi-slice helical computed tomography was requested in order to evaluate the insertion of the ribs in the sternum (Figure 1).

**Figure 2.**
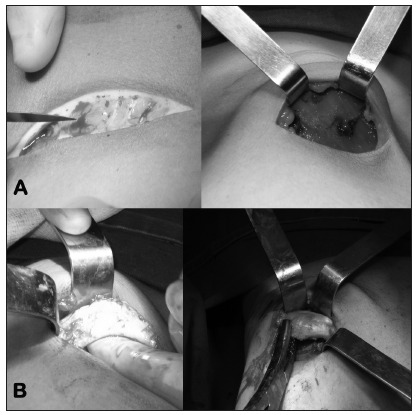
Surgical procedure. A) Incision. B) Resection of the rib.

The surgical procedure consisted of bilateral incisions in the inframammary crease and dissection of subcutaneous soft tissue and intercostal muscles to expose the chondral insertion of the eighth, ninth and tenth ribs into the sternum. The thoracic team dissected the periosteum and removed the excess chondral cartilage and a segment of the body of the ribs, large enough to correct the chest protrusion (Figure 2). Through the same incision, the plastic surgery team dissected the subcutaneous tissue over the pectoralis major and inserted a subglandular polyurethane breast implant (235 ml).

The idea of performing both procedures using a single bilateral incision was shown to be feasible and safe, and it allowed the scar to be minimally visible.

The procedure was performed without intercurrences. During the postoperative period, an epidural catheter was used to provide analgesia. The patient was discharged on the third day after the surgery, without the analgesia catheter.

**Figure 3.**
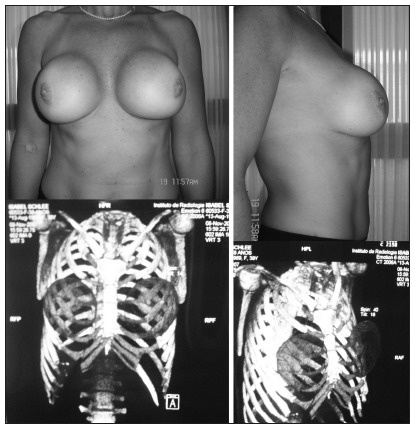
Photographs of the patient and multi-slice helical computed tomography two years after the operation.

The technique of correcting the defect of the anterior thoracic wall was found to be effective. The patient’s thorax and breasts have now an appropriate shape, as demonstrated two years later (Figure 3). Correction through a single incision benefited the patient and provided a satisfactory esthetic result.

## DISCUSSION

Reports about treatments for chest wall deformities appear frequently in the medical literature, but there have only been a few reports of esthetic surgical treatment for patients with mammary ptosis and deformities of the chest wall (Table 1).

**Table 1.** Results from our review of the medical databases using descriptors for the main clinical findings observed in our patient

Database	Search strategy (MeSH and DeCS)	Results*
Medline (PubMed)	#1 “Thoracic wall deformities” AND “breast implants”	3 case reports
	#2 “Augmentation mammoplasty” and “thoracic wall deformities”	1 case report
	#3 “Pectus” OR “chest deformity”	940 articles
	#4 “Congenital Abnormality” OR “congenital defects” AND “thorax or chest”	44 articles
Scirus	#1	1 case report
	#2	-
	#3	10 articles
	#4	20,341 articles
Cochrane Library	#1	-
	#2	-
	#3	16 (4 reviews, 9 clinical trials, 1 method study, 1 technology assessment and 1 economic evaluation)
	#4	20 (4 reviews and 16 clinical trials)
Lilacs Databases	#1	-
	#2	-
	#3	60 articles
	#4	-
Embase	#1	-
	#2	-
	#3	-
	#4	-

The misfortune of having a deformity such as *pectus excavatum* may influence most phases of psychological and physical development. According to Einsiendel,^5^ the psychological effects are more severe after the age of 11 years, when intensified feelings such as duress, social anxiety, shame, negativism, intolerance, frustration and even depression may appear.^5^

Adequate psychological treatment contributes towards reestablishing such patients’ mental health, thereby rehabilitating them to normal social relations and providing relief to their families.^6^ In view of the great psychological torment caused by such deformities, surgeons need to be aware of the formative processes of bones and cartilages in the thoracic wall and their etiology and pathogenesis, along with the treatment options that exist. It is also necessary to be familiar with the various kinds of deformities.^7^

Mammary ptosis may provoke all the psychological effects mentioned above. For this condition, surgical treatment with breast implants would be an appropriate alternative because of its low morbidity, easy execution and satisfactory results. Insertion of silicone prosthesis breast implants has shown good results, independently of the point of access, the dissection plane or the nature of the prosthesis.^8^

The literature shows different possibilities for the surgical indications and the methods for surgical treatment for *pectus excavatum* (implant placement, sternochondroplasty or fat transplantation).^9^

Surgeons need to bear in mind that breast defects are often related to anterior thoracic wall defects in women. Hence, satisfactory results depend on correction of both deformities. In view of the esthetic issues involved in correcting thoracic deformities, and in breast cancer surgery, techniques that can minimize the surgical scars should be preferred.^10^^,^^11^^,^^12^^,^^13^

## CONCLUSION

This technique for repairing mammary ptosis and a moderate type of *pectus excavatum*, performed by the plastic and thoracic surgery teams, was shown to be effective for correcting both deformities. Cooperation between these teams was fundamental for this treatment. The result presented a good esthetic and functional effect, with consequent reestablishment of the patient’s self-esteem.
